# Effective regulation of technology in women’s health and healthcare

**DOI:** 10.1136/bmj.r2351

**Published:** 2025-11-07

This article by Sara Raza and colleagues (*BMJ* 2025;391:e086300; doi:10.1136/bmj-2025-086300, 10 October 2025) mentioned unintended pregnancies associated with use of the Natural Cycles app. It has been amended to clarify that the failure rate of the app was within the expected range.

